# Transcriptomic Analysis of Canine Osteosarcoma from a Precision Medicine Perspective Reveals Limitations of Differential Gene Expression Studies

**DOI:** 10.3390/genes13040680

**Published:** 2022-04-13

**Authors:** Rebecca L. Nance, Sara J. Cooper, Dmytro Starenki, Xu Wang, Brad Matz, Stephanie Lindley, Annette N. Smith, Ashley A. Smith, Noelle Bergman, Maninder Sandey, Jey Koehler, Payal Agarwal, Bruce F. Smith

**Affiliations:** 1 Scott-Ritchey Research Center, College of Veterinary Medicine, Auburn University, Auburn, AL 36849, USA; rln0005@auburn.edu (R.L.N.); xzw0070@auburn.edu (X.W.); agarwpa@auburn.edu (P.A.); 2 Department of Pathobiology, College of Veterinary Medicine, Auburn University, Auburn, AL 36849, USA; mzs0011@auburn.edu (M.S.); jaw0007@auburn.edu (J.K.); 3 HudsonAlpha Institute for Biotechnology, Huntsville, AL 35806, USA; sjcooper@hudsonalpha.org (S.J.C.); dstarenki@irepertoire.com (D.S.); 4 Center for Advanced Science, Innovation, and Commerce, Alabama Agricultural Experiment Station, Auburn, AL 36849, USA; 5 Department of Clinical Sciences, College of Veterinary Medicine, Auburn University, Auburn, AL 36849, USA; bmm0007@auburn.edu (B.M.); ses0034@auburn.edu (S.L.); smith30@auburn.edu (A.N.S.); aas0042@auburn.edu (A.A.S.); nsb0009@auburn.edu (N.B.)

**Keywords:** osteosarcoma, transcriptome, sequencing, cancer, canine

## Abstract

Despite significant advances in cancer diagnosis and treatment, osteosarcoma (OSA), an aggressive primary bone tumor, has eluded attempts at improving patient survival for many decades. The difficulty in managing OSA lies in its extreme genetic complexity, drug resistance, and heterogeneity, making it improbable that a single-target treatment would be beneficial for the majority of affected individuals. Precision medicine seeks to fill this gap by addressing the intra- and inter-tumoral heterogeneity to improve patient outcome and survival. The characterization of differentially expressed genes (DEGs) unique to the tumor provides insight into the phenotype and can be useful for informing appropriate therapies as well as the development of novel treatments. Traditional DEG analysis combines patient data to derive statistically inferred genes that are dysregulated in the group; however, the results from this approach are not necessarily consistent across individual patients, thus contradicting the basis of precision medicine. Spontaneously occurring OSA in the dog shares remarkably similar clinical, histological, and molecular characteristics to the human disease and therefore serves as an excellent model. In this study, we use transcriptomic sequencing of RNA isolated from primary OSA tumor and patient-matched normal bone from seven dogs prior to chemotherapy to identify DEGs in the group. We then evaluate the universality of these changes in transcript levels across patients to identify DEGs at the individual level. These results can be useful for reframing our perspective of transcriptomic analysis from a precision medicine perspective by identifying variations in DEGs among individuals.

## 1. Introduction

Osteosarcoma (OSA) is a highly aggressive and spontaneous tumor of the bone seen primarily in the appendicular skeleton of pediatric patients. Approximately 10–20% of patients exhibit macro-metastatic lesions at the time of diagnosis, while 80–90% of patients are presumed to harbor micro-metastases [1]. Metastases occur almost exclusively in the lungs and, once present, make management difficult. The 5-year survival rate for cases with detectable metastasis at the time of diagnosis is approximately 20–25% [1,2]. The current standard-of-care treatment includes surgical resection and combination chemotherapy [1]. However, 30–40% of patients will relapse within 3 years of starting treatment [3]. Metastectomy is considered the second-line treatment and improves 5-year survival rates to 40% [4]. Moreover, osteosarcoma is particularly proficient in acquiring multiple drug-resistant pathways, a major limiting factor for patient survival. Subsequent reoccurrence is exceedingly common and repeated systemic chemotherapy is frequently required; unfortunately, the eventual resistance of the metastases to treatment is inevitable [4]. Despite significant advances in the cancer therapy domain, the standard-of-care treatment and survival rates for OSA have remained essentially unchanged for 40 years [1,5,6]. For these reasons, it is imperative to explore and implement improved models to develop more effective treatment approaches.

Canine OSA has the potential to serve as an excellent model for the human disease due to similar clinical, histological, and molecular characteristics [7,8,9]. Primary tumors occur at similar sites with comparable histological presentation, response to treatment, and occurrence and distribution of metastases [10]. Further support for dogs as a valuable translational model is evidenced by a cross-species gene signature study which used oligonucleotide arrays to examine expression from a limited set of orthologous genes. The researchers found indistinguishable gene expression patterns between canine and pediatric OSA [7]. Studies utilizing whole genome and/or whole exome sequencing have reached similar conclusions [8,9]. The primary difference between canine and human OSA is the age of onset. Canine OSA has a bimodal age distribution, with peaks at 18–24 months and 7 years, though older, larger breed dogs are typically affected more often. On the other hand, human OSA primarily occurs in the second decade of life [4]. OSA prevalence is also greater in dogs, occurring 27 times more frequently than in humans, and progression occurs rapidly [11]. With amputation of the affected limb in combination with chemotherapy, the 1-year survival rate for dogs is approximately 45% [11].

New molecular tools, such as next-generation sequencing, have allowed significant improvements to be made in the identification of common genetic changes that are associated with specific types of cancer [12]. This technology has inaugurated the era of personalized or precision medicine, which utilizes an individual’s specific genetic changes to guide treatment. This approach seeks to classify individual patients into groups based on the presence of key gene mutations that directly impact the tumor’s sensitivity to specific chemotherapeutic agents. In this manner, treatments can be selected that have a higher likelihood of efficacy due to a better understanding of the relevant functioning pathways in that particular tumor.

At the molecular level, OSA, whether human or canine, is characterized by substantial genetic complexity and instability [13]. Relapsed OSA is considerably more complex, involving multiple drug-resistant pathways. The unique genetic complexity of OSA poses limitations for therapy, and precision medicine is not immune to these limitations [14]. The personalized treatment of two high risk human OSA patients based on comprehensive molecular profiling via next-generation exome sequencing showed no significant benefit to overall health or disease progression [15]. However, the targeted therapy was implemented after chemotherapy and refractory disease appeared, when drug resistance mechanisms are highly convoluted and make interpretation difficult. Fortunately, precision medicine is now entering a new phase where the transcriptomic analysis of individual patients may provide unique perspectives for treatment by addressing the intrinsic heterogeneity of gene expression in tumors, both within and between patients. A common approach to identifying targetable components of a tumor involves the analysis of differentially expressed genes (DEGs) in tumors compared to normal tissue.

Patient samples and clinical information are important factors to consider in differential gene studies and are unfortunately often overlooked. The source of normal tissue should be derived from the tumor’s cell-of-origin, and, in the case of OSA, that includes osteoblasts and osteocytes. Prior studies of differential gene expression in OSA have used RNA isolated from a canine osteoblast cell line [9]; tissues from unrelated organs such as liver, lymph node, and kidney [7]; tissue harvested adjacent to the tumor, potentially jeopardizing the normality of the sample [16]; bone tissue from unrelated patients [17]; or have not clearly described the origin of the matched normal samples [18,19]. While these data are undoubtedly useful to advancing our understanding of the disease, evaluation using primary normal bone cells harvested distally from the tumor within the same patient may yield more appropriate results. Furthermore, consistency in chemotherapy status among patients at the point of sample collection is important for drawing conclusions related to tumor status. The administration of chemotherapeutic agents provides a selective pressure that alters the tumor’s cellular population and phenotype [14,20]. Analysis of tumor gene expression prior to any chemotherapy provides a snapshot of the tumor phenotype, independent of acquired drug resistance or variation in individual drug response. Many studies in human OSA have utilized tissues harvested from individuals after the onset of chemotherapy, making interpretation of the results difficult [15,21,22].

DEG analysis combines the tumor and normal samples into two distinct groups to derive statistically meaningful DEGs that are generalizable across individuals. However, combining patients into a group implies that the samples are similar, if not identical, thereby contradicting and disregarding the intra- and inter-tumoral heterogeneity that forms the basis of precision medicine. While this type of analysis is critical for determining statistical differences in the group, individual-level analysis can provide additional insight into the differences among patients.

Given these limitations of traditional group differential gene expression studies, we supplemented this analysis with a more novel approach of evaluating individual tumors based on genes identified in the group approach. This study uses transcriptomic sequencing of RNA derived from seven primary canine osteosarcoma tumors with patient-matched normal bone to identify DEGs and pathways. Importantly, the normal bone samples, which serve as the comparator for determining baseline gene expression levels, were harvested from the phalanges of each patient. Furthermore, all samples were obtained prior to chemotherapy or evidence of macrometastatic lung disease. We explore the resulting DEGs in terms of group analysis for tumor vs. bone, as well as the discrete analysis of individual patients to identify the extent of heterogeneity in gene expression among individuals. These results highlight the phenotypic diversity of primary OSA among individuals and provide a supplemental approach to traditional methods of analyzing DEGs, particularly when the goal is an application in precision medicine.

## 2. Materials and Methods

### 2.1. Description of Data

Animals: Samples were obtained from seven dogs undergoing routine limb amputation for the clinical treatment of osteosarcoma (OSA) at the Auburn University College of Veterinary Medicine. Patient characteristics are briefly summarized in Table 1. The histopathology of tissue immediately adjacent to that used for RNA extraction was performed to confirm OSA and ensure that intact neoplastic tissue was entered into the experimental pipeline. Thoracic radiographs performed prior to amputation indicated no evidence of pulmonary macro-metastatic disease. In all cases, samples were obtained prior to chemotherapy treatment.

### 2.2. RNA Isolation and Sequencing

Normal bone RNA: Patient-matched samples were collected to obtain normal bone RNA to allow transcript levels to be compared with the tumors. The second phalanx was removed from the amputated leg within one hour of amputation. In all cases, the tumor was at least one joint space proximal to the phalanx. Due to the location, which was distal and distant from the primary tumor, it is unlikely that the normal bone sample had undergone any neoplastic events related to the tumor. For each dog, bone preparation and RNA extraction for the phalanx samples was performed according to Nance et al. [23].

Tumor RNA: For each dog, the tumor was dissected into approximately 3 mm by 3 mm samples, flash frozen in liquid nitrogen, and stored at −80 °C until RNA extraction. RNA isolation was accomplished using a homogenizer in combination with acid guanidinium thiocyanate-phenol-chloroform extraction. After weighing, approximately 100–200 mg of the frozen tumor samples was added to a 14 mL snap cap tube (Falcon) containing 2 mL of chilled Tri-Reagent (Molecular Research Center, Cincinnati, OH, USA). The sample was subjected to mechanical homogenization in short but frequent bursts until sufficiently homogeneized, as determined visually. The samples were divided into two microcentrifuge tubes and after 10 min incubation at room temperature, 100 μL of bromochloropropane (BCP) (Molecular Research Center) was added to each, vortexed thoroughly, and incubated for 5 min at room temperature. After centrifugation at 20,000× *g* for 15 min at 4 °C, the RNA-containing aqueous layer was carefully transferred to a new tube. Contaminating DNA was removed by the addition of DNase I Reaction Buffer (10% of the total volume) and 10 μL DNase I (Thermo Fisher Scientific Waltham, MA, USA), followed by a 10 min incubation at room temperature. DNase was inactivated by the addition of 10 μL of EDTA and heated at 65 °C for 10 min. Following this, 10% volume sodium acetate (3 M, pH 5.2) (VWR International, Radnor, PA, USA) was added to each tube, followed by 70% volume isopropyl alcohol, and the samples were briefly vortexed. Following a 15-min incubation at −20 °C, the samples were centrifuged at 20,000× *g* for 20 min at 4 °C. The supernatant was decanted, the pellet dislodged in 1 mL 70% ethanol, and centrifuged at 20,000× *g* for 30 min at 4 °C. After the supernatant was removed, the pellet was air dried for approximately 5–10 min and subsequently resuspended in 15 μL of RNase-free water. The RNA was quantified using a Nanodrop 2000 instrument (Thermo Fisher Scientific).

RNA Sequencing: The samples were commercially prepared and sequenced (HudsonAlpha Institute for Biotechnology, Huntsville, AL, USA). Initial quality control analysis was performed using the Agilent Bioanalyzer 2100 (Agilent Technologies, Santa Clara, CA, USA) and an RNA integrity number (RIN) was generated. RNA sequencing libraries were produced with 500 ng of total RNA using the TruSeq PolyA library kit (Illumina) to deplete samples of any RNA aside from polyadenylated mRNA. They were pooled and sequenced on two lanes of the Illumina HiSeq v4 (PE, 50 bp, 25 M reads) yielding an average of 34 M reads per sample.

### 2.3. Bioinformatic Pipeline and Data Analysis

**Figure 1:**
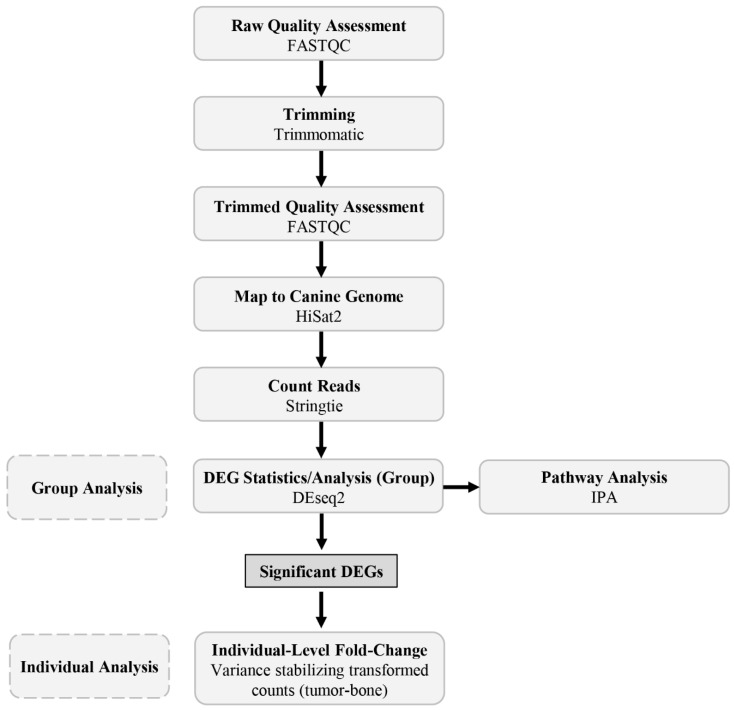
Overview of bioinformatic pipeline used to process and analyze data. RNA sequence data were subjected to modest trimming with Trimmomatic and quality analysis with FASTQC prior to mapping to the reference canine genome with HiSat2, counting reads with Stringtie, and DEG statistics with DEseq2 to generate significant DEGs (FDR < 0.05, FC > 2, ≤2) which were carried forward in pathway and individual analyses. Using the significant DEGs from the group analysis, fold-change values were generated for each patient to produce the individual-level data. Bioinformatic tools and packages utilized are indicated.

The trimming of adapters and the first leading base was performed using Trimmomatic (v0.40) [24] with a minimum length of 36 bp and raw quality was assessed using FASTQC (v0.10.1) [25]. After trimming, approximately 32–45 million sequence reads remained for each sample and FASTQC was used to ensure all bases had a Phred quality score above 28. Reads were mapped to the indexed canine reference genome (CanFam3.1) obtained from ENSEMBL (release 103) using HiSat2 (v2.2.1) [26] and a table of mapped read counts was generated with Stringtie (v1.3.3) [27]. On average, 93% of the reads mapped to the canine reference genome. The bioinformatic pipeline is summarized in Figure 1.

### 2.4. Group Analysis

The statistical analysis and identification of differentially expressed genes was performed using DEseq2 (v3.14) [28] to generate the traditional group analysis results (Supplementary Data S1). These results provide a list of genes that are broadly differentially expressed among samples. The DEseq2 package applies a general linear model with a negative binomial distribution and applies the Benjamini–Hochberg procedure to control for the false discovery rate (FDR). The pre-filtering of genes with less than 1 read was performed prior to statistical analysis. A multi-factor design was used for statistical analysis to include patient ID as a term in the design formula (design = ~dog + tissue source). This design has been recommended in the DEseq2 vignette for analyzing paired samples because it accounts for differences between individuals. Log2 fold-changes were calculated relative to bone. To extract significant DEGs while minimizing noise, the data were filtered using a false discovery rate (FDR) less than 0.05, base mean greater than 10, and log2 fold-change greater than 1 and less than −1 (corresponding to a fold-change of 2 and −2, respectively) (Supplementary Data S2). The variance stabilizing transformation function in DEseq2 was used to transform the data to fit an approximately homoscedastic distribution and to remove the dependence of the variance on the mean [28]. The results of this transformation were used for the visualization and clustering of the results to generate a heatmap and a principal component analysis plot. Hallmark pathway analysis and gene ontology (GO) enrichment analysis of the upregulated genes was performed using Metascape with a *p*-value cut-off of 0.01, minimum overlap of 3, and minimum enrichment of 1.5 [29]. Due to the constraints of gene nomenclature in Metascape, only the upregulated genes with identified human orthologs (total 670) were included in the analysis. Raw counts are available in the Supplementary Information (Supplementary Data S4).

### 2.5. Individual Analysis

To explore how the DEGs varied among individual patients, we first filtered the data to include only the significant genes as defined by FDR < 0.05, base mean > 10, and an FC cut-off of 2 from the classical group analysis. We then calculated log2 fold-change values for each gene by subtracting the variance-stabilized transformed counts (on the log2 scale) of bone from tumors for each dog (Supplementary Data S3). We used these results to determine the number one upregulated and downregulated gene in each patient.

## 3. Results

### 3.1. RNA Quality

RIN numbers were used to determine sequencing suitability based on RNA integrity. Samples with an RIN below 5 cannot guarantee reliable sequencing results; for all except two (patient B bone RIN = 4.5, patient A tumor RIN = 5.0), the RIN was above 5, indicating moderately intact RNA. The average RIN for tumor and bone was 6.8 and 6.5, respectively.

### 3.2. Group Analysis

**Figure 2:**
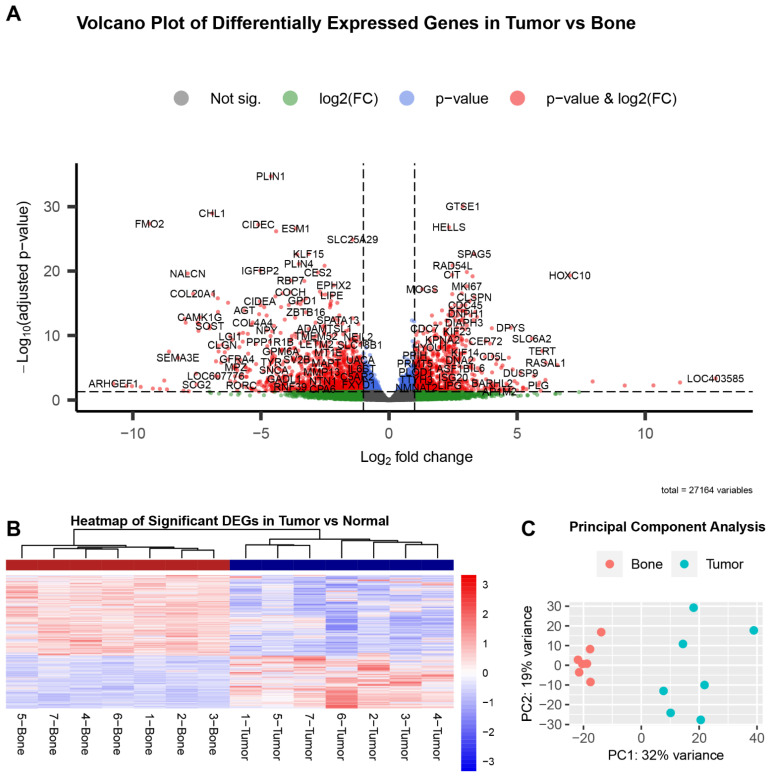
Analysis of differential gene expression in tumor vs normal group data reveals over 3000 significant DEGs. Volcano plot shows many genes are highly differentially expressed between tumor vs. normal (**A**). Genes indicated in red are significant in terms of both adjusted *p*-value (<0.05) and fold-change (>2 and ≤2). The sign of fold-change (positive or negative) was retained and is reported in terms of tumor compared to bone. Heatmap of the significant genes (FDR < 0.05) shows that these DEGs easily differentiate tumor from normal tissue (**B**). Each row represents a gene and upregulation is indicated in red, while downregulation is shown in blue. Patient and sample ID is indicated underneath the corresponding column. PCA plot shows grouping of normal bone samples (red circles) distinct from tumor samples (blue circles) as expected (**C**). The dispersion and variability of the tumor samples is thought to be related to intra- and inter-tumoral heterogeneity.

For the traditional group analysis, we compared the gene expression from all tumor tissues to all normal tissues and found a total of 3742 differentially expressed genes with a false discovery rate (FDR) of less than 0.05. After further filtering using a base mean greater than 10 and fold-change values greater than 2 and less than −2, there were 2031 significant DEGs. Of these, 803 genes were upregulated with a fold-change greater than 2, and 1228 genes were downregulated with a fold-change less than −2 in tumor compared to normal bone (Figure 2A). Hierarchical clustering of the 2031 significant DEGs shows that the normal and tumor samples cluster together in terms of over- and under-expression (Figure 2B). This is as expected based on studies in other organisms where as many as a third of genes show evidence for altered expression in tumors compared to normal tissue.

Principal component analysis was performed to further evaluate sample clustering (Figure 2C). As expected, all bone samples clustered together, distinct from the tumor samples.

To evaluate the biologically significant DEGs, we subset the significant results based on a fold-change cut-off of 2 and sorted them based on the log2 fold-change or adjusted *p*-value (FDR). Ordering based on log2 fold-change allows us to evaluate the most highly over- or under-expressed genes that still meet the adjusted *p*-value below 0.05. On the other hand, ordering the gene list based on an adjusted *p*-value provides DEGs with higher confidence in shared trends among individuals. The top 10 downregulated and upregulated genes are listed in Table 2. Many of the canine ENSEMBL gene identifications did not correspond to known gene symbols or human orthologs for the top 10 genes when ordered based on smallest and largest log2 fold-change, but when ordered based on smallest adjusted *p*-value, all 10 genes corresponded to known gene symbols (Table 2). Based on sorting by padj, the top upregulated genes included GTSE1, HELLS, SPAG5, RAD54L, and IQGAP3. The top downregulated genes included PLIN1, CL1, FMO2, CIDEC, and ESM1.

**Figure 3:**
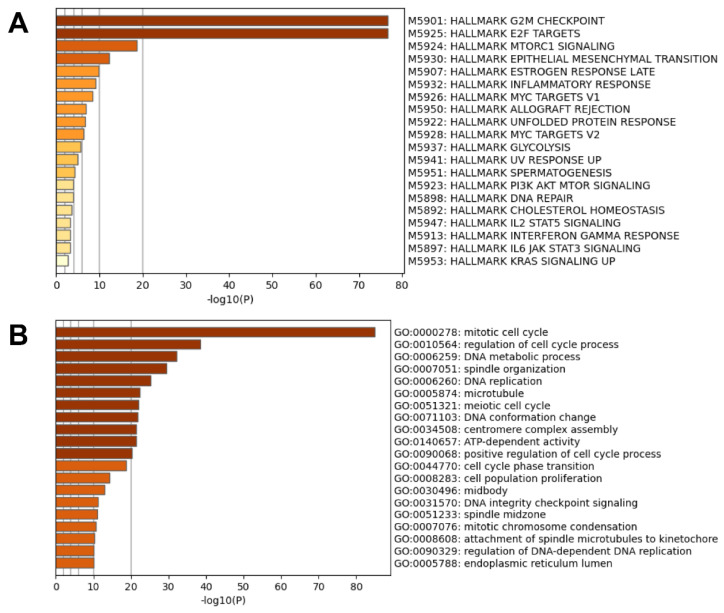
Pathway analysis of the upregulated DEGs using hallmark pathways (**A**) and gene ontology (GO) enrichment terms (**B**).

Hallmark pathway analysis of the upregulated DEGs revealed G2M checkpoint (M5901), E2F targets (M5925), MTORC1 signaling (M5924), and epithelial mesenchymal transition (M5930) as the most enriched pathways in tumors (Figure 3A). The top pathways from gene ontology (GO) enrichment analysis included the mitotic cell cycle (GO:0000278), regulation of cell cycle process (GO:0010564), DNA metabolic process (GO:0006259), and spindle organization (GO:0007051) (Figure 3B). These results are consistent with previously published data.

**Figure 4:**
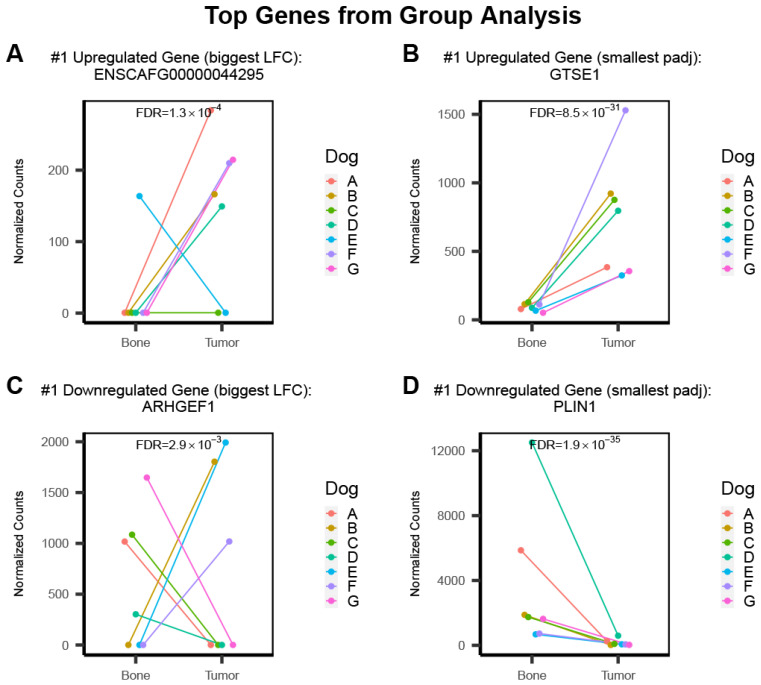
Plots of the normalized counts of the top up- and down-regulated genes in the classical group analysis show some variation among individual patients. The number one gene when ordered by log2 fold-change shows variation among individual patients (**A**,**C**). The top gene when ordered by smallest adjusted *p*-value shows less variation in terms of direction of fold-change, but some variation between individuals is still evident (**B**,**D**).

The classical group analysis identifies DEGs that are broadly dysregulated among patients. As a whole, each of the tumors exhibited similar patterns in terms of DEGs and these are distinct from normal bone. However, expression was not necessarily consistent across patients for all DEGs. To observe how the top genes for the group analysis varied between individuals, we plotted the normalized counts of the number one upregulated and downregulated gene according to the smallest adjusted *p*-values (Figure 4B,D) and the largest log2 fold-change difference (Figure 4A,C). The top upregulated gene according to the largest fold-change difference was ENSCAFG00000044295 (L2FC = 13.5, FDR = 1.3 × 10^−4^), which, according to ENSEMBL, encodes an uncharacterized protein of 120 amino acids in length. The top upregulated gene with the smallest FDR was GTSE1 (L2FC = 2.9, FDR = 8.6 × 10^−31^), or G2 and S phase-expressed protein 1, which encodes a protein that binds the tumor suppressor protein P53 to repress its ability to induce apoptosis in response to DNA damage. The top downregulated gene according to fold-change was ARHGEF1 (L2FC = −10.7, FDR = 2.9 × 10^−3^), or rho guanine nucleotide exchange factor 1, which encodes a protein that may be involved in forming a complex with G proteins and the stimulation of rho-dependent signals. The top downregulated gene with the smallest FDR was PLIN1 (L2FC = −4.6, FDR = 1.9 × 10^−35^), or perilipin 1, which encodes a protein involved in adipocyte lipid metabolism.

Visualization of these normalized gene counts shows some variation among patients, some even showing an inverse relationship despite having an FDR < 0.05 (Figure 4C).

### 3.3. Individual Analysis

**Figure 5:**
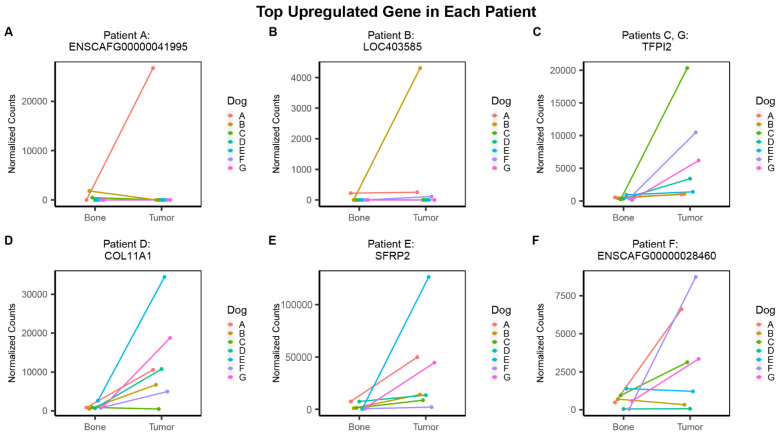
Plots of the top upregulated gene for each dog based on individual-level analysis reveal further variation among patients. The normalized counts of the top upregulated gene in each patient is shown. The top upregulated gene for patient A was ENSCAFG00000041995 (**A**). The top upregulated gene for patient B was LOC403585 (**B**). Patients C and G shared the same top upregulated gene, TFPI2 (**C**). The top upregulated gene for patient D was COL11A1 (**D**). The top upregulated gene for patient E was SFRP2 (**E**). The top upregulated gene for patient F was ENSCAFG00000028460 (**F**).

To supplement the classical group analysis, we sought to identify the top DEGs in each individual patient by calculating log2 fold-change values for each pair of tumor and normal samples using the significant genes from the group analysis. Figure 5 depicts the normalized counts of the top upregulated gene in each patient. While the results show a general trend shared among individuals (Figure 5C–F), the top genes for patient A and B are heavily skewed by substantial counts in the tumor of that specific transcript, and minimal to no counts in the other patients (Figure 5A,B). The top upregulated gene for patient A, ENSCAFG00000041995, encodes a predicted long non-coding RNA of 1359 bp with unknown functions (Figure 5A). Interestingly, this gene was found to be the second top downregulated gene when ordered by fold-change for the group analysis, with a log2 fold-change of −10.25 and FDR of 0.006 (Table 2). For patient B, the top upregulated gene was LOC403585, also known as serum amyloid A1, which encodes a protein that is expressed in response to inflammation (Figure 5B). In the group analysis, this gene was found to be the second top upregulated gene when ordered by fold-change, with a log2 fold-change of 12.75 and an FDR of 0.0004. The top upregulated gene for both patients C and G was TFPI2 (tissue factor pathway inhibitor 2), which encodes a serine proteinase inhibitor that has been identified as a tumor suppressor in a variety of cancers (Figure 5C). The top upregulated gene in patient D, COL11A1 (collagen type XI α 1 chain), encodes a protein component of pro-collagen type XI, a major component of bone tissue (Figure 5D). For patient E, the top upregulated gene was SFRP2 (secreted frizzled related protein 2), which encodes a protein involved in Wnt signaling (Figure 5E). The top upregulated gene in Patient F was ENSCAFG00000028460, which encodes two long non-coding RNA transcripts with unknown functions (Figure 5F). These three genes, COL11A1, SFRP2, and ENSCAFG00000028460, were not observed in the top 20 down- or up-regulated genes from the group analysis.

**Figure 6:**
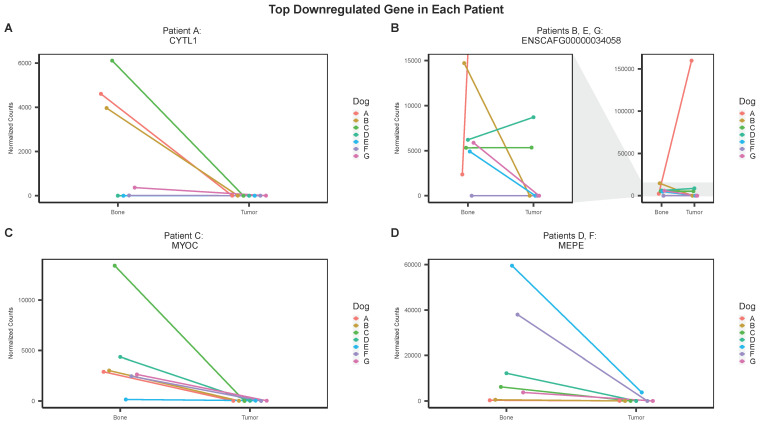
The top downregulated gene for each individual dog is shared among some patients. The normalized counts of the top downregulated gene in each patient are shown. The top downregulated gene in patient A was CYTL1 (**A**). Patients B, E, and G share the same top downregulated gene, ENSCAFG00000034058 (**B**). The top downregulated gene in patient C was MYOC (**C**). Patients D and F share the same top downregulated gene, MEPE (**D**).

Similarly, the top downregulated gene for each patient was determined and more overlap among individuals was observed (Figure 6). The top downregulated gene for patient A was CYTL1 (cytokine-like 1), which encodes a protein that is expressed in bone marrow and cord blood mononuclear cells with the CD34 surface receptor [30] (Figure 6A). The top downregulated gene for patients B, E, and G was ENSCAFG00000034058, which encodes a 1501 bp long non-coding RNA with uncharacterized function (Figure 6B). The top downregulated gene for patient C was MYOC (myocilin) which encodes a protein involved in cytoskeletal function (Figure 6C). The top downregulated gene in patients D and F was MEPE (matrix extracellular phosphoglycoprotein) which encodes a protein component of the extracellular matrix of bone (Figure 6D).

To compare the individual and group analysis results, we extracted the log2 fold-change values and adjusted *p*-values (FDR) from the group analysis for each top gene from the individual analysis (Table 3). While the results show similarities, the top upregulated gene in patient A (ENSCAFG00000041995) shows distinct opposition in terms of the direction of fold-change from the group analysis (L2FC = −10.25). In fact, while this gene is significantly over-expressed in patient A’s tumor, it is modestly downregulated in two tumors (patients B and C) and shows no evidence of expression in tumor or normal bone for the remaining patients (Figure 5A). Furthermore, this gene was identified as the second most downregulated gene from the group results when ordered by log2 fold-change (Table 2).

## 4. Discussion

Osteosarcoma is a highly complex and volatile bone tumor primarily seen in pediatric patients for which treatment has remained stagnant for almost 40 years. It is well-established that canine OSA is an ideal model for studying the human disease, as well as developing and testing therapeutic alternatives, such as precision medicine concepts. Using dogs, we aim to fill gaps in the current knowledge of OSA tumorigenesis while identifying potential therapeutic targets that can be tested and evaluated in a clinical setting.

While the technology of precision medicine has rapidly advanced, the overall clinical efficacy of such therapies leaves much to be desired, due primarily to the inherent heterogeneity and rapid evolution of drug-resistant mechanisms that define cancer [31]. Though cancer is traditionally considered a disease of genotypic origin, the phenotype reflects the accumulated genetic complexity and actionable targets that may be exploited. Transcriptomic sequencing provides a snapshot of gene expression and a link between genotypic and phenotypic landscape. In the context of cancer, where aberrant transcriptional patterns are pervasive, transcriptome profiling can identify and quantify changes in gene activity that are distinctive of tumors. Using this information, we can identify differentially expressed genes associated with various cellular processes and pathways that may be broadly expressed among patients to guide current treatment options and develop new therapies.

Traditional differential gene analysis groups individuals together to derive statistically meaningful DEGs; however, this approach fails to account for intra- and inter-tumoral heterogeneity and therefore is limited in its application for precision medicine. Deriving individual fold-change values provides additional insight into the differences among patients and potential targets that may be beneficial for a subset of patients. Individual-level analyses are difficult due to the lack of statistical inference that can be derived from a single sample. To address this limitation, we selected only the significant DEGs from the group analysis to be carried forward to the individual-level analysis, thereby providing some statistical basis for the individual results. However, it must be recognized that some distinctive gene expression alterations may be so specific to one individual that they will be lost from this analysis.

Using this concept, we sequenced the transcriptomes of seven primary canine OSA tumors and patient-matched normal bone samples to derive differentially expressed genes (DEGs). In contrast to previously published studies, all samples were collected before chemotherapy and evidence of macro-metastatic lung disease to limit confounding interpretation of the results. Furthermore, normal bone samples were collected from each patient to generate suitable baseline gene expression levels. To our knowledge, this is the first study to derive normal expression for each patient using a biologically appropriate sample (bone) which is representative of the tumor’s cell-of-origin (osteoblasts and osteocytes, depleted of bone marrow) in primary osteosarcoma prior to chemotherapy. This pairwise comparison reduces biological variability, increases statistical power, and provides a more thorough perspective of differential gene expression.

The clustering of the DEGs derived from the traditional group analysis showed distinct relationships between tumor and normal samples as a group. The wide dispersion and variability in the tumor samples, as evidenced in the PCA plot (Figure 2C), is believed to be related to the intra- and inter-tumoral heterogeneity. The bone tissues were depleted of bone marrow and any cells other than those embedded within the bone matrix, whereas the tumor samples may contain many different types of cells at various stages of differentiation (for example, infiltrating immune cells and de-differentiated cells with a more stem-cell-like phenotype). Furthermore, even tumors of the same type or source exhibit heterogeneity in their molecular profile [32].

We used traditional group analysis to statistically identify the top genes that are broadly dysregulated among the patients. Significant DEGs were selected based on padj < 0.05, FC > 2 or <−2, and a base mean count > 10, and these results were ranked based on log2 fold-change as well as an adjusted *p*-value. The top upregulated genes from the group analysis included GTSE1 (when sorted by padj) and ENSCAFG00000044295 (when sorted by log2 fold-change). GTSE1 regulates the G1/S cell cycle transition and has been reported to be overexpressed in other human cancers [33,34,35,36]. GTSE1 has been implicated in conferring cisplatin resistance in human osteosarcoma, though more studies are necessary to confirm its role in OSA tumorigenesis [37]. ENSCAFG00000044295 encodes a 120 amino acid protein with uncharacterized functions that does not correlate to any human ENSEMBL IDs. The top downregulated genes included PLIN1 (when sorted by padj) andARHGEF1 (when sorted by log2 fold-change). The downregulation of PLIN1 mRNA has been reported in breast cancer and is considered a tumor suppressor in breast cancer progression [38,39]. The dysregulation of rho GTPases, including ARHGEF1, are commonly reported in a variety of cancers [40].

We showed that the group results can sometimes be misleading due to the heterogeneity among individuals. In some cases, a bi-directional change in expression was observed among patients. To circumvent this, we used individual-level analysis to derive log2 fold-change values for each patient using only the significant genes identified in the group analysis. We used this information to identify the top upregulated and downregulated genes in each patient and compare their expression across individuals. The top downregulated gene was shared between patients B, E, and G (ENSCAFG00000034058), as well as for patients D and F (MEPE). ENSCAFG00000034058 encodes a long non-coding RNA with unknown functions. Co-expression network analysis has revealed MEPE as a dysregulated gene in human osteosarcoma [41]. The top upregulated gene was different for all individuals (A: ENSCAFG00000041995, B: LOC403585, D: COL11A1, E: SFRP2, F: ENSCAFG00000028460), except for patients C and G (TFPI2). The upregulation of COL11A1 and SFRP2 has been reported in human osteosarcoma [22,42]. TFPI2, a tumor suppressor gene, has been shown to be upregulated in other cancers, including colorectal, gastric, and prostate [43,44,45]. Both ENSCAFG00000041995 and ENSCAFG00000028460 encode uncharacterized long non-coding RNAs that do not correlate to known human ENSEMBL gene IDs. More studies are needed to evaluate their role in OSA tumorigenesis.

The top upregulated gene in patient A (ENSCAFG00000041995) showed an inverse relationship in terms of the direction of fold-change in the group analysis. In fact, this gene was the second-most downregulated gene according to the group results sorted by log2 fold-change, despite having an adjusted *p*-value less than 0.05. The observed differences in gene expression among individuals may be due to (1) the effects of different mutations in combination with genetic and/or environmental effects, (2) the stage of the tumor, or (3) initial RNA quality. This example represents the hallmark conclusion of this study; despite relatively stringent filtering conditions to minimize noise and confidently identify the top DEGs, traditional group analyses can be misleading and lead to the identification of therapeutic targets that may be ineffective for most patients.

Consistent with the conventional theme of research, this study is not without limitations. With a small sample size of seven, the statistical power is limited. As with all next-generation sequencing studies, computational challenges may limit the downstream interpretation. For example, the extent of gene annotation in the canine reference genome (CanFam3.1) limited our ability to identify the appropriate human orthologs. Additionally, it is becoming increasingly evident that non-coding RNAs such as micro-RNA and long non-coding RNA (lncRNA) play a dynamic role in tumor progression [18,19]. This study utilized a sequencing approach that excluded RNAs without poly-A tails. However, some lncRNAs are produced and processed with poly-A tails and as such were included in the sequencing. Furthermore, the heterogeneity of tumors makes it difficult to understand and characterize the phenotypic landscape, its components, and how those components affect tumor progression. It is also difficult to identify the distribution of these changes; for example, a highly upregulated gene may be vastly over-expressed, but only prevalent in only a small subset of cells. The clonal hypothesis implies a spatial distribution of different tumor cell populations, and using a single small section for sequencing may impair our ability to detect other cellular subsets. With the advancement of single-cell RNA sequencing and the development of accompanying bioinformatics tools, we may be able to elucidate the details of a tumor’s phenotype more clearly. While more studies are needed to evaluate the role these dysregulated genes play in tumorigenesis, these results support the notion that traditional group DEG analysis should be supplemented by individual-level analysis in the context of precision medicine for the identification of potential therapeutic targets.

## 5. Conclusions

Canine OSA serves as an excellent model for determining molecular targets and developing and evaluating personalized precision treatment. The relatively ample availability of dogs with spontaneously occurring OSA provides a powerful and underused translational model. Limb amputation offers the opportunity to easily collect tumor samples for sequencing as well as appropriate normal tissue, for example, from the distal phalanges, to allow comparisons within the same patient prior to chemotherapy [23].

The goal of precision medicine is to use an individual tumor’s molecular profile, rather than tumor category or stage, to inform therapeutic decisions and design novel treatments. Group DEG analysis has been traditionally used to identify relevant genes, but these results do not account for intra- or inter-tumoral differences. Despite using a multi-factor paired design to account for differences between individuals, as well as strict filtering parameters to minimize noise, our results showed some conflicting elements and variation in DEG expression. These results suggest that identifying significantly up- or downregulated genes as potential therapeutic targets using traditional group analysis may not always be appropriate, even when stringent filtering conditions are used. While these conclusions may be well established in the bioinformatics community, they may be lesser known in the precision oncology field, where DEG analysis is pervasive. Supplementing DEG analyses with individual-level analyses provides additional insight into the intra- and inter-tumoral heterogeneity.

The novelty of this study lies in the sample set as well as the analytical workflow. In contrast to other published studies, (1) all samples were obtained prior to chemotherapy, which can alter the phenotype; (2) normal bone samples (depleted of contaminating tissue) from each patient were used as the comparator tissue for baseline gene expression; and (3) DEG analysis was supplemented by individual-level analysis and compared to group DEG analysis. This study demonstrates that the variation in DEG expression between individuals, obtained using traditional group DEG analysis, is sufficient to warrant further individual-level analysis to identify more effective targets for precision therapy.

## Figures and Tables

**Table 1:** Summary of canine osteosarcoma patients. Tumor and patient-matched normal bone samples were obtained from seven dogs undergoing limb amputation for osteosarcoma. Age is based on the date of limb amputation and breed is reported per the owner. (RIN = RNA integrity number, yrs = years, mos = months).

Patient	Sex	Neuter Status	Age	Tumor Site	Breed	Tumor RIN	Bone RIN
A	M	Castrated	10 yrs, 6 mos	Left distal radius	Golden Retriever	5.0	6.6
B	M	Castrated	7 yrs	Left distal femur	Rottweiler	7.4	4.5
C	M	Castrated	7 yrs	Left distal radius	Doberman Pinscher	6.9	6.5
D	M	Castrated	11 yrs, 9 mos	Left proximal humerus	Greyhound	6.4	6.7
E	F	Spayed	7 yrs, 6 mos	Left distal radius	Great Pyrenees	6.9	6.9
F	M	Castrated	7 yrs	Right distal radius	Golden Retriever	8.3	7.1
G	F	Spayed	9 yrs, 9 mos	Right distal tibia	Greyhound	6.7	7.2

**Table 2:** List of top 10 upregulated and downregulated genes in tumor vs. normal from group analysis. When ordered by lowest and highest log2 fold-change, many of the ENSEMBL gene identifications do not correspond to known gene symbols. When ordered by smallest adjusted *p*-value (FDR), all ENSEMBL IDs correspond to known symbols.

Top 10 Downregulated Genes in Tumor, Ordered by Log2 Fold-Change				
ENSEMBL	SYMBOL	Log2FC	Padj	GENE NAME
ENSCAFG00000028799	ARHGEF1	−10.75	2.93 × 10^−3^	Rho guanine nucleotide exchange factor 1
ENSCAFG00000041995	NA	−10.25	6.33 × 10^−3^	NA
ENSCAFG00000007622	NA	−10.04	6.95 × 10^−3^	NA
ENSCAFG00000005350	NA	−9.69	9.07 × 10^−3^	NA
ENSCAFG00000014986	FMO2	−9.35	4.40 × 10^−28^	flavin containing dimethylaniline monoxygenase 2
ENSCAFG00000042006	NA	−9.22	1.63 × 10^−2^	NA
ENSCAFG00000049609	NA	−8.94	1.84 × 10^−2^	NA
ENSCAFG00000008109	NA	−8.77	9.28 × 10^−4^	NA
ENSCAFG00000029213	LOC607979	−8.67	2.22 × 10^−2^	eukaryotic translation initiation factor 3, subunit L pseudogene
ENSCAFG00000011465	NA	−8.59	3.20 × 10^−8^	NA
**Top 10 Upregulated Genes in Tumor, Ordered by Log2 Fold-Change**				
ENSCAFG00000044295	NA	13.49	1.31 × 10^−4^	NA
ENSCAFG00000009135	LOC403585	12.75	4.34 × 10^−4^	serum amyloid A1
ENSCAFG00000032019	NLRP12	11.36	1.85 × 10^−3^	NLR family pyrin domain containing 12
ENSCAFG00000013213	NA	10.32	5.31 × 10^−3^	NA
ENSCAFG00000043115	NA	9.20	5.53 × 10^−3^	NA
ENSCAFG00000007173	NA	7.95	1.31 × 10^−3^	NA
ENSCAFG00000006648	HOXC10	7.06	4.67 × 10^−3^	homeobox C10
ENSCAFG00000015211	APOBEC3Z1	6.58	3.81 × 10^−6^	apolipoprotein B mRNA editing enzyme, catalytic polypeptide-like
ENSCAFG00000008986	RASAL1	6.12	1.68 × 10^−6^	RAS protein activator like 1
ENSCAFG00000035513	LOC111093651	6.11	7.53 × 10^−4^	uncharacterized LOC111093651
**Top 10 Downregulated Genes in Tumor, Ordered by Adjusted *p*-value**				
ENSCAFG00000011986	PLIN1	−4.61	1.91 × 10^−35^	perilipin 1
ENSCAFG00000006248	CHL1	−6.90	1.11 × 10^−29^	cell adhesion molecule L1 like
ENSCAFG00000014986	FMO2	−9.35	4.40 × 10^−28^	flavin containing dimethylaniline monoxygenase 2
ENSCAFG00000005266	CIDEC	−5.10	6.25 × 10^−28^	cell death inducing DFFA like effector c
ENSCAFG00000018381	ESM1	−3.63	2.38 × 10^−27^	endothelial cell specific molecule 1
ENSCAFG00000006392	ACKR4	−4.41	6.62 × 10^−27^	atypical chemokine receptor 4
ENSCAFG00000030764	SLC25A29	−1.42	1.31 × 10^−25^	solute carrier family 25 member 29
ENSCAFG00000003807	KLF15	−3.14	2.28 × 10^−23^	Kruppel-like factor 15
ENSCAFG00000001854	AQP7	−3.58	2.66 × 10^−23^	aquaporin 7
ENSCAFG00000015323	PLIN4	−3.52	7.60 × 10^−22^	perilipin 4
**Top 10 Upregulated Genes in Tumor, Ordered by Adjusted *p*-value**				
ENSCAFG00000000782	GTSE1	2.88	8.51 × 10^−31^	G2 and S-phase expressed 1
ENSCAFG00000008090	HELLS	2.34	1.54 × 10^−27^	helicase, lymphoid specific
ENSCAFG00000018724	SPAG5	3.33	2.28 × 10^−23^	sperm associated antigen 5
ENSCAFG00000004272	RAD54L	2.48	1.35 × 10^−21^	RAD54 like
ENSCAFG00000016616	IQGAP3	3.04	1.41 × 10^−20^	IQ motif containing GTPase activating protein 3
ENSCAFG00000010114	CIT	2.46	3.84 × 10^−20^	citron rho-interacting serine/threonine kinase
ENSCAFG00000006648	HOXC10	7.06	4.67 × 10^−20^	homeobox C10
ENSCAFG00000016090	TOP2A	3.26	6.47 × 10^−20^	DNA topoisomerase II α
ENSCAFG00000013255	MKI67	3.04	2.35 × 10^−18^	marker of proliferation Ki-67
ENSCAFG00000008478	MOGS	1.30	6.47 × 10^−18^	mannosyl-oligosaccharide glucosidase

**Table 3:** Comparison of individual-level analysis with group analysis results. The top up- and downregulated gene for each patient according to log2 fold-change values from individual analysis shows some similarities to group analysis results. The top upregulated gene in patient A (ENSCAFG00000041995) shows distinct opposition in terms of direction of fold-change in the group results.

		Patient	Gene	Log2FC (Individual)		Log2FC (Group)	FDR
Individual Analysis Results	Top Upregulated Gene	A	ENSCAFG00000041995	9.03	Group Analysis Results	−10.25	6.39 × 10^−3^
		B	LOC403585	6.42		12.75	4.37 × 10^−4^
		C	TFPI2	5.92		3.23	3.60 × 10^−6^
		D	COL11A1	3.81		3.18	1.49 × 10^−9^
		E	SFRP2	8.63		3.19	7.36 × 10^−6^
		F	ENSCAFG00000028460	6.09		1.79	4.15 × 10^−2^
		G	TFPI2	4.68		3.23	3.60 × 10^−6^
	Top Downregulated Gene	A	CYTL1	−6.13		−6.65	2.34 × 10^−4^
		B	ENSCAFG00000034058	−8.17		−5.92	3.31 × 10^−2^
		C	MYOC	−8.04		−7.97	2.58 × 10^−13^
		D	MEPE	−7.33		−7.47	1.06 × 10^−12^
		E	ENSCAFG00000034058	−6.61		−5.92	3.31 × 10^−2^
		F	MEPE	−9.05		−7.47	1.06 × 10^−12^
		G	ENSCAFG00000034058	−6.86		−5.92	3.31 × 10^−2^

## Data Availability

The data used in this study have been deposited into the NCBI GEO repository under accession number GSE199489. Data and scripts used for processing and generating figures can be found at DEseq2, https://github.com/rln0005/OSA_RNAseq (accessed on 1 February 2022).

## Supplementary Materials

The following supporting information can be downloaded at: https://www.mdpi.com/article/10.3390/genes13040680/s1, Data S1: Results for all genes, Data S2: significant genes, Data S3: individual fold-change, Data S4: raw gene counts.
